# PacBio amplicon sequencing for metabarcoding of mixed DNA samples from lichen herbarium specimens

**DOI:** 10.3897/mycokeys.53.34761

**Published:** 2019-06-03

**Authors:** Cécile Gueidan, John A. Elix, Patrick M. McCarthy, Claude Roux, Max Mallen-Cooper, Gintaras Kantvilas

**Affiliations:** 1 Australian National Herbarium, National Research Collections Australia, CSIRO-NCMI, Canberra, ACT, 2601, Australia Australian National Herbarium Canberra Australia; 2 Research School of Chemistry, Building 137, Australian National University, Canberra, ACT, 2601, Australia Australian National University Canberra Australia; 3 64 Broadsmith St, Scullin, ACT, 2614, Australia Unaffilaited Canberra Australia; 4 390 chemin des Vignes vieilles, 84120 Mirabeau, France Unaffilaited Mirabeau France; 5 Centre for Ecosystem Science, School of Biological, Earth and Environmental Sciences, University of New South Wales Sydney, Kensington, NSW, 2052, Australia University of New South Wales Sydney Sydney Australia; 6 Tasmanian Herbarium, Tasmanian Museum and Art Gallery, Sandy Bay, Tasmania 7005, Australia Unaffiliated Hobart Australia

**Keywords:** SMRT sequencing, high-throughput sequencing, long amplicon analysis (LAA), lichenised fungi

## Abstract

The detection and identification of species of fungi in the environment using molecular methods heavily depends on reliable reference sequence databases. However, these databases are largely incomplete in terms of taxon coverage, and a significant effort is required from herbaria and living fungal collections for the mass-barcoding of well-identified and well-curated fungal specimens or strains. Here, a PacBio amplicon sequencing approach is applied to recent lichen herbarium specimens for the sequencing of the fungal ITS barcode, allowing a higher throughput sample processing than Sanger sequencing, which often required the use of cloning. Out of 96 multiplexed samples, a full-length ITS sequence of the target lichenised fungal species was recovered for 85 specimens. In addition, sequences obtained for co-amplified fungi gave an interesting insight into the diversity of endolichenic fungi. Challenges encountered at both the laboratory and bioinformatic stages are discussed, and cost and quality are compared with Sanger sequencing. With increasing data output and reducing sequencing cost, PacBio amplicon sequencing is seen as a promising approach for the generation of reference sequences for lichenised fungi as well as the characterisation of lichen-associated fungal communities.

## Introduction

Across the world, herbaria and fungal culture collections host large numbers of specimens and strains with the main goal being to document, preserve and classify the many species within the fungal kingdom. Among characters generally used to identify fungi, DNA sequences have been particularly useful for the numerous fungal species with plastic or convergent morphologies. Of the DNA markers traditionally used for fungal identification, the internal transcribed spacer region (ITS) was chosen as the primary barcode because it allowed species-level identification for the broadest range of fungi (Schoch et al. 2012). Following the formal acceptance of this fungal barcode, substantial effort was put into improving the curation of ITS sequences in several databases, including RefSeq (Schoch et al. 2014; O’Leary et al. 2015), UNITE (Kõljalg et al. 2013; Nilsson et al. 2018) and ISHAM-ITS (Irinyi et al. 2015), by selecting high-quality ITS sequences generated from reliably identified and preserved specimens. These reference databases play a critical role in the sequence-based species identification of herbarium specimens, cultured strains and fungal communities from environmental samples (Tedersoo and Nilsson 2016). However, linking sequences to names remains a challenge, partly because of the incompleteness of these reference datasets (Orock et al. 2012; Kõljalg et al. 2013; Crous et al. 2014; Nilsson et al. 2018). The molecular barcoding of herbarium specimens, including generic and species types therefore remains a priority (Crous et al. 2014; Yahr et al. 2016).

Although the characterisation of fungal diversity in environmental samples has benefited immensely from the development of next generation sequencing (NGS) technologies, the high-throughput generation of reference ITS sequences from herbarium specimens has been hindered by the short length of Illumina reads, the most commonly used NGS platform. With a size ranging from 500 up to 1200 bp, the full length of the ITS region cannot be sequenced as a single DNA fragment. For DNA extractions of lichen specimens, which harbor on their surface or within their thalli a plethora of other fungi, as well as for any other DNA samples of mixed fungal communities, assembling DNA markers from a pool of short reads belonging to several species carries a high risk of obtaining chimeric sequences (Hebert et al. 2018). As a consequence, fungal metabarcoding studies mostly use only half of the ITS region, either ITS1 or ITS2 (Nilsson et al. 2010; Mello et al. 2011; Blaalid et al. 2013). The generation of full length and high-quality barcodes from lichen specimens for reference databases or identification purposes generally involved Sanger sequencing (e.g., Kelly et al. 2011; Orock et al. 2012; Leavitt et al. 2014; Divakar et al. 2016; Xu et al. 2017). However, this method faces a similar challenge: that co-amplified non-lichenised fungal sequences often prevent the generation of readable target sequences (Kelly et al. 2011; Orock et al. 2012). Of the few available methods to separate sequences from target and non-target species (e.g., gel separation, group-specific primers), cloning has proven to be the most broadly applicable method for obtaining high quality sequences of the target lichenised fungus in mixed samples (Hofstetter et al. 2007). It does, however, significantly increase the cost and time required for generating high-quality sequences.

Long-read sequencing allows us to circumvent these challenges. For lichenised fungi, early long amplicon sequencing studies made use of Roche 454 pyrosequencing technology, either for the full ITS region (Hodkinson and Lendemer 2013; Mark et al. 2016) or for ITS1 only (Lücking et al. 2014). In Mark et al. (2016), with a target sequence recovered for 99 of the 100 samples studied, the application of this technology for lichen specimen metabarcoding seemed promising. However, the development of this sequencing technology was abandoned by Roche and is now obsolete. Long read sequencing using single molecule real-time (SMRT) sequencing technology (Pacific Biosciences, USA) is becoming more affordable, and has recently been applied to fungi for metabarcoding purposes (Chen et al. 2015; Cline and Zak 2015; James et al. 2016; Schlaeppi et al. 2016; Walder et al. 2017; Heeger et al. 2018). Some of the challenges usually associated with SMRT sequencing (high error rate, high rate of chimeric formation and high cost per sample) have been investigated and partly addressed. The high error rate of SMRT raw reads (about 15%; Goodwin et al. 2016) is significantly decreased due to the multiple passes obtained from a single polymerase read using circularised amplifications (Travers et al. 2010), as well as the correcting nature of high sequence coverage for randomly distributed errors (Koren et al. 2012). The error rate of circular consensus sequences (CCS) is now usually under 1% (Goodwin et al. 2016), and compares well with other sequencing platforms, including Illumina (Schlaeppi et al. 2016; Schloss et al. 2016) and Sanger (Goodwin et al. 2016). Using mock communities, the rates of formation of chimeric sequences (up to 16.3% in Heeger et al. 2018) were shown to be in the same range as the one obtained with short read sequencing (D’Amore et al. 2016). As for the cost per sample, it can be reduced thanks to multiplexing (Heeger et al. 2018) to a level where the cost of DNA extraction or PCR amplification becomes higher than the sequencing cost (Hebert et al. 2018).

The scarcity of automated pipelines to analyse SMRT amplicon data for different applications, including community analysis, remains an issue (Heeger et al. 2018; Tedersoo et al. 2018). Most software developed for sequencing-error correction and sequence assembly have been optimised for short reads and have not been evaluated for SMRT raw data (Heeger et al. 2018). During the SMRT sequencing primary analysis, image processing, base-calling and quality assessment are done in real-time on the instrument. Polymerase reads are then used to generate CCSs, which, in addition to the raw polymerase read files, are provided by the sequencing facility as fasta or fastq files. For community analyses, CCSs can then be analysed by various software or software packages, such as Mothur (Schloss et al. 2009) or PipeCraft (Anslan et al. 2017), in order to demultiplex, filter, cluster and assign consensus sequences to a taxonomic unit (see Anslan et al. 2018). Although PacBio (Pacific Biosciences) provides secondary analysis modules as part of the SMRT portal, none of these is specifically designed for community analysis. The Long Amplicon Analysis (LAA) pipeline available on SMRT Link and SMRT Portal attracted our interest, as it identifies differing clusters of sequencing reads within a single library and is capable of differentiating between underlying sequences that are 99.9% similar, such as haplotypes and pseudogenes (Bowman et al. 2014). The main application of LAA is allele phasing and detection in diploid organisms (Bowman et al. 2014; Lleras et al. 2014) and it works as follows. Error-corrected subreads (CCS) obtained from polymerases reads are first demultiplexed (samples are separated using unique molecular indexes or combinations of indexes), then filtered depending on read quality and length. They are then aligned (overlap step) and clustered based on the alignment. Each cluster is iteratively phased in order to separate high-scoring mutations (alleles). Resulting sub-clusters are polished using a Quiver-based method to produce high-quality consensus sequences, which then go through a last filtering step to detect and remove PCR artefacts (e.g., chimeric sequences). This pipeline was designed to differentiate sequencing errors from true sequence variations, allowing the preservation of all sequence variants within a sample. For each index/sample barcode, the output includes all unique consensus sequences obtained, together with their coverage value and predicted accuracies. LAA can be performed on the instrument’s SMRT portal, allowing the sequencing provider to send these demultiplexed consensus sequences directly to the customer, saving time and effort in software installation and raw data analyses.

In this study, the use of SMRT sequencing and the LAA pipeline for the production of ITS barcode sequences from lichen herbarium specimens was explored. The goals were: 1) to establish a high-throughput protocol to obtain indexed PCR products from lichen DNA extracts for SMRT sequencing; and 2) to investigate the ability of LAA to recover high-quality sequences for the target lichenised fungal species as well as for non-target fungal species.

## Material and methods

### DNA extractions

A total of 96 specimens were selected for their frequent need for cloning as well as their relevance to several ongoing taxonomic studies on Australian lichens at the Australian National Herbarium (see Suppl. material 1: Table S1). The genera represented were *Catillaria* (41 specimens from Australia and 16 from France), *Buellia* (39 specimens from Australia), *Endocarpon* (8 specimens from Australia) and *Verrucaria* (2 specimens from France). The specimens were 1–34 years old and are kept at CANB, MARSSJ and in the private herbarium of M. Bertrand. For crustose specimens (*Catillaria*, *Buellia* and *Verrucaria*), material (thallus and fruiting bodies) was detached from the substrate with a clean single-edge razor blade and a folded sheet of weigh paper was used to collect and transfer the material to a tube (8-strip cluster tubes, Corning Incorporated, Salt Lake City, USA). For *Endocarpon*, squamules were detached from the soil substrate using clean tweezers and transferred directly to a tube.

Each of the 96 cluster tubes contained a washed chrome steel bearing ball 3 mm in diameter (3MMCH/S/B grade 40, BSC Bearing & Power Transmission Solutions, Chullora, Australia). The samples were ground with a TissueLyser II (Qiagen, Hilden, Germany) in two cycles of 1 min and a frequency of 25/s. The cluster tubes were centrifuged for 1 min at 6,000 rpm and the caps were removed with care to avoid cross-contaminations. A lysis buffer was added to each tube. Genomic DNA was extracted using the Invisorb® DNA Plant HTS 96 kit (STRATEC Molecular, Berlin, Germany) following the manufacturer’s instructions, except for the last centrifugation step which was changed to 10 min at 2,000 rpm (instead of 5 min at 4,000 rpm) to avoid breaking the elution plates. A 1/10 dilution of the DNA extraction plate was prepared and subsequently used for amplification.

### Amplification, normalisation and pooling

Indexed PCR products were generated using the PacBio Barcoded Universal Primers protocol (https://www.pacb.com/wp-content/uploads/2015/09/Procedure-and-Checklist-Preparing-SMRTbell-Libraries-PacB-Barcoded-Universal-Primers.pdf). The ITS barcode (internal transcribed spacer 1, 5.8S ribosomal RNA subunit and internal transcribed spacer 2) was the region targeted. With a first PCR, our target region was amplified using the primers ITS1F (Gardes and Bruns 1993) and ITS4 (White et al. 1990), both modified by adding a 5’ block and a tail representing the PacBio universal sequences. In a 25 µl reaction, 5 µl of buffer, 1 µl of MyFi (Bioline, London, UK), 1 µl of 5 µM of each primer, 16 µl of water and 1 µl of DNA template (1/10 dilution) were added. The 96 PCR reactions were performed in strip tubes with individual caps to avoid cross-contaminations. The PCR programme was 5 min at 95 °C, then 20 cycles of 30 sec at 95 °C, 30 sec at 53 °C and 1:30 min at 72 °C, followed by a final elongation step for 7 min at 72 °C. Four strips were randomly selected and their PCR products run on to an agarose gel using the nucleic acid stain GelRed (Biotium, Fremont, CA, USA). Because the gel showed primer dimer bands in addition to the amplicon bands, the PCR products were cleaned using Sera-Mag magnetic beads (SpeedBead Magnetic Carboxylate Modified Particles, GE Healthcare) and a 96 well Alpaqua® magnetic plate. The cleaning was done by adding 0.8× volume of beads to each PCR product, followed by two washes with 200 µl of 70% ethanol. Dry beads were then resuspended in 25 µl of the elution buffer from the Invisorb® DNA Plant HTS 96 kit.

A second amplification was then performed using the Barcoded Universal F/R Primers Plate-96 available from PacBio (Millenium Science, Mulgrave, VIC, Australia). In a 25 µl reaction, 5 µl of buffer, 1 µl of MyFi, 2.5 µl of the barcoded primers, 15.5 µl of water and 1 µl of the product of the first round of PCR were added. The PCR programme was 3 min at 95 °C, then 20 cycles of 30 sec at 95 °C, 30 sec at 53 °C and 1:30 min at 72 °C, followed by a final elongation step for 7 min at 72 °C. All PCR products were checked on a gel as previously described. For samples showing no band, a new round of amplification (first PCR with ITS tailed primers and second PCR with barcoded universal primers) was performed using the non-diluted DNA extracts. Positive PCR products generated by this second round of amplification were used to substitute the negative samples on the original plate. The samples for which neither the dilution nor the original extract gave amplicons were left on the original plate. The 96 samples were cleaned as described above and their concentration measured with a Nanodrop 8000 spectrophotometer (Thermo Scientific, Waltham, MA, USA). All samples with a DNA concentration larger than 50 ng/µl were normalised manually to 50 ng/µl. One microliter of each of the 96 samples was pooled into a 1.5 ml Eppendorf tube.

### Library preparation, sequencing and SMRT Portal primary analysis

The pooled sample (1.5 µg of DNA) was sent to the Ramaciotti Centre for Genomics (UNSW Sydney, Australia) for single molecular real-time (SMRT) sequencing. The sample met the quality control requirements and showed two clear peaks at 650 and 923 bp, which are within the expected size range for the ITS barcode. The library preparation was done using the PacBio Barcoded Universal Primers protocol (https://www.pacb.com/wp-content/uploads/2015/09/Procedure-and-Checklist-Preparing-SMRTbell-Libraries-PacB-Barcoded-Universal-Primers.pdf). The sample was sequenced in one SMRT cell on a PacBio RSII using a P6 chemistry with a four-hour movie. The raw data were analysed using Long Amplicon Analysis (LAA v. 1) with barcoding option on the SMRT Portal. This pipeline was run using the following settings: symmetric DNA barcodes with a minimum score of 22, a minimum subread length of 450 bp, a maximum number of subreads of 4,000, and default values for all other parameters. The phase alleles option was selected.

### Molecular identification and validation

After demultiplexing, quality control and generation of consensus sequences using LAA, the amplicon data was provided in 96 files in fasta format. Sequences from the targeted lichenised fungal species were identified from fungal endophytes or contaminants based on sequence similarity using a BLASTN v 2.8.1 search on the nr database by excluding uncultured/environmental sample sequences (https://blast.ncbi.nlm.nih.gov/; search done on Dec 12–14 2018). The first BLASTN match (or highest bit score) was recorded unless it did not correspond to at least a fungal Class, in which case the next match was considered. For 12 samples, the target sequence obtained with PacBio was cross-validated by a sequence obtained with Sanger sequencing. The ITS barcode was amplified with the primers ITS1F and ITS4 using the DNA polymerase MyFi as previously described, and products were sent to Macrogen (Seoul, Korea) for purification and sequencing. PacBio and Sanger sequences were manually compared in Mesquite v 3.51 (Maddison and Maddison 2017). For samples for which multiple ITS barcodes were recovered for the target species, the sequence versions were compared using Mesquite and the BLASTN suite-2 sequences was used to estimate percentage identity between selected sequence pairs. Sequences with the highest coverage were selected as primary barcodes. Secondary barcodes were only taken into consideration when their coverage was above 30 and when they were less than 98% similar from the primary barcode. Raw PacBio files and FASTQ files of primary and secondary barcodes obtained were deposited in the Sequence Read Archive on NCBI (BioProject ID PRJNA541190).

## Results

### Amplifications

After the two amplification steps, 19 of the 96 samples did not show any visible product. For these samples, a new round of amplifications was performed using the non-diluted DNA extracts instead of the 1/10 dilution. This second amplification round generated products for 13 samples that were previously negative. These additional products were then used to substitute the corresponding negative samples on the original plate. For the five samples for which none of the amplification rounds was positive (sample barcode numbers 41, 45, 73, 84, 92, 93), the initial sample were not substituted and were pooled together with the positive samples for final submission.

### Sequencing

A first SMRT cell was run with a 0.02 nM sample, but resulting ZMW (Zero-Mode Waveguide) productivity was poor (P1=12%). After filtering, only 17,582 polymerase reads were recovered for this first run, which corresponded to 247,626 subreads (Table 1). The quality of the resulting subreads was, however, acceptable (mean of 14.68 passes). A second SMRT cell was then used with a 0.05 mM concentration of the same sample. The second run showed good ZMW productivity (P1=55%). After filtering, 82,501 polymerase reads were recovered (Table 1). The number of post-filter subreads obtained from this run was 1,095,489 and their quality was comparable to the first run (mean of 13.79 passes). Subreads from both SMRT cell runs were combined and analysed with the Long Amplicon Analysis protocol in the SMRT portal. After demultiplexing, the number of pre-filter subreads per barcode ranged from 91–115,311 (Suppl. material 1: Table S1). Barcodes with low subread numbers (<200) mostly corresponded to samples with negative (41, 45, 73, 93) or weak (53) amplification. The number of CCS obtained for each barcode ranged from 1–948. No chimeric sequences were detected in any of the barcoded samples, but a few low-quality sequences (noise) were found. After clustering and phasing, 0–32 final consensus sequences were obtained per barcode, with a length of 619–2,322 bp, a coverage of 8–500 (the default value of the maximum number of subreads to cluster is 500) and a predicted accuracy of 0.9525–0.9999. The predicted accuracy of the most represented sequence per barcode was above 0.9999 for the majority of the samples (93%).

**Table 1.** Productivity and sequence output from two SMRT runs with different loading concentration.

Loading concentration	ZMW productivity			Post-filter polymerase reads			Subreads		
	P0	P1	P2	number	mean length	N50	number	mean length	number of passes
0.02 nM	86%	12%	2%	17,582	15,234 bp	27,799 bp	247,626	1038 bp	14.68
0.05 nM	29%	55%	16%	82,501	15,321 bp	21,997 bp	1,095,489	1111 bp	13.79

### Sequence identity

Sequences were recovered for 89 of the 96 samples (see Suppl. material 1: Table S1). For each positive sample, 1–32 sequences were obtained using LAA. A BLASTN search was used to identify the sequence of the target species (the lichenised fungi of interest) among the co-amplicons. Eighty-five samples had at least one blast hit that matched the target species. The search revealed that, despite no detection by the PacBio pipeline LAA, a few chimeric sequences (about 3% of the total number of obtained sequences) were obtained, seemingly consisting of two or three full and/or partial sequences, often of the same species, occurring in tandem. Samples for which either no sequence (e.g., samples 41 and 45) or no target species sequences (e.g., samples 92 and 95) principally derived from amplification reactions with no or weak PCR products. Some reactions with weak products did generate a sequence for the target species, although generally with a coverage lower than 100 (e.g., samples 29, 94 and 96).

For 46 samples, a single sequence was obtained for the target species among all the co-amplified fungal taxa. For the other samples, the clustering and phasing process resulted in 2–9 sequences that could be attributed to the target species using the BLASTN match. The similarity between these different sequence versions was investigated in an alignment. After reverse complement and barcode trimming, these sequence versions were in fact identical for an additional ten samples, bringing the total of successful target single-sequences to 56. A few sequence versions also differed with indels in long single nucleotide repeats (e.g., samples 27, 27, 28, 49, 64, 68, 69) and were the likely result of poor quality for low coverage consensus sequences. For the remaining samples, differences between sequence versions were more random (indels and single nucleotide mutations) and more likely due to genuine sequence heterogeneity within a sample, either due to biological reasons (concerted evolution or mixed individual) or carry-over contaminations. The percentage identity between the most divergent target sequence versions of each sample ranged between 94.78 and 99.98% (Suppl. material 1: Table S1).

In addition to the target species, sequences were also obtained for other fungi for 81 of the 96 samples. The great majority of these co-amplifying fungal sequences (479 out of 506 sequences) belonged to the ascomycetes, with Capnodiales and Chaetothyriales being the most commonly represented orders. Several sequences (23) belonged to the basidiomycetes, two to the chytridiomycetes and one to the mucoromycetes. Finally, one sequence matched *Pirulasalina*, a Xanthophyceae algae from the order Tribonematales.

### Validation

Sanger sequences were obtained for 12 samples. For five of these (3, 6, 8, 61 and 76), the Sanger sequence was entirely identical to the PacBio sequence. For five additional samples (5, 7, 58, 60 and 74), the Sanger sequence was identical to the PacBio sequence, but contained ambiguous bases or was slightly shorter due to missing bases at the 5’ or 3’ ends. For two samples (4 and 57), the Sanger sequences comprised one nucleotide difference compared to the PacBio sequences in addition to several ambiguous bases. These differences were located at the start or the end of the sequence and were likely due to poor quality chromatograms in the 5’ and 3’ regions of the Sanger sequences.

## Discussion

Collections of well-curated lichen specimens are important resources for species identification, whether done traditionally using morpho-anatomical together with chemical characters, or using DNA barcodes. For the latter, access to databases with high quality sequence data, detailed voucher information and reliable taxon names is critical. Although current reference sequence datasets exist, they are still incomplete. Thus, the molecular barcoding of lichen specimens for a broad range of species remains a priority (Yahr et al. 2016). In this study, a SMRT sequencing metabarcoding approach generated ITS sequences for 85 of the 96 lichen specimens investigated. This study shows that for lichen herbarium specimens up to 25 years old, full length and high-quality ITS sequences can be recovered for the target lichenised fungus using PacBio amplicon sequencing. Furthermore, sequences were also obtained for co-amplifying fungi, shedding light on the diversity of endolichenic fungi in the samples.

The protocol used in this study enabled the high-throughput processing of samples and the bioinformatic pipeline permitted the recovery of high-quality sequences with minimum time and effort, because both the primary and secondary analyses could be carried out by the sequencing provider. For mixed lichen DNA extracts, SMRT sequencing is a cheaper option than cloning and Sanger sequencing when high number of samples need to be processed. The cost of producing a barcode Sanger sequence is generally around AUD$15/sample (including DNA extraction, amplification and sequencing costs), but when cloning is required, this cost can increase to AUD$100/sample. With the PacBio amplicon protocol used in this study and the PacBio RSII platform, the cost per sample was estimated at AUD$37. This cost could be further reduced by co-amplifying more markers and/or multiplexing more samples. With the new PacBio platform Sequel, multiplexing 384 samples for one marker would bring the cost down to AUD$12/sample. Moreover, with the recent increased output per SMRT cell (from 1 gigabase for RSII to 20 gigabases for the new PacBio platform Sequel), the cost per sample could fall even further with higher multiplexing levels.

The use of PacBio amplicon sequencing for high-throughput barcoding of lichen specimens is therefore very promising. Some challenges remain, both for the laboratory and the bioinformatic aspects of this method. Some considerations and suggestions for improvements based on this study are outlined below, especially with regard to the preparation of the amplicon library and the generation of ITS barcode sequences using the LAA pipeline.

### Technical considerations for high-throughput DNA extraction and amplicon library preparation

A relatively high risk of carry-over contaminations is generally associated with two-step PCR amplicon protocols (Seitz et al. 2015), including the PacBio Barcoded Universal Primers protocol used in this study. In a preliminary test of this protocol (data not shown), output sequences suggested a significant number of potential carry-over contaminations. In light of these results, particular care was taken at various stages of the final run when handling the genomic DNA and PCR samples. More specifically, DNA extracts were stored in single tubes as opposed to a 96-well plate, and amplifications were carried out in strip tubes with individual caps instead of 96-well plates. In the final SMRT run, carry-over contaminations may have also occurred, but at a much lower level than the preliminary test run. Our current protocol could therefore be improved further. More specifically, the use of a PCR hood or post- and pre-PCR separation, as previously recommended (Urban et al. 2000; Jones and Kustka 2017), would be advisable for this two PCR step protocol. During DNA extraction, the use of 8-strip cluster tubes for the grinding of material should be replaced by single tubes, as opening the lids of these tubes causes a potential risk for sample cross-contamination. Minimising the number of pipetting steps and the opening of tubes after amplification is also important. In our protocol, a PCR clean-up step was required after the first PCR due to strong PCR dimer bands. Optimisation of primer concentrations in order to reduce primer dimer formation would eliminate this step. Finally, the inclusion of several replicates of the same sample within a SMRT run would facilitate the assessment of replicability between samples and potential carry-over contaminations.

The choice of polymerase has been shown to be important when using HT sequencing for rare allele detection (Hestand et al. 2016; Potatov and Ong 2017) as well as for community analysis (Oliver et al. 2015), because it impacts directly on the rate of PCR-induced errors. Polymerases with proofreading activities are usually recommended for library preparation protocols, including for SMRT amplicon sequencing. High-fidelity polymerases are, however, more expensive and, in our experience, are more difficult to optimise. A possible incompatibility between a high-fidelity polymerase and SMRT sequencing has even been suggested previously (Schlaeppi et al. 2016). As argued by Hebert et al. (2018), for the molecular barcoding of reference specimens, the dominant sequence will match the source sequence even when a relatively high number of other sequences have PCR errors. In our preliminary test of this protocol, only a small number of PCR products could be recovered using the high-fidelity Phusion Hot Start II (Thermo Scientific). As the focus of our study was to obtain a barcode sequence from the target species, and not to characterise fungal communities using sequences, the polymerase MyFi was used to generate amplicons. It is marketed as being efficient with challenging templates and inhibitor-rich samples and has so far worked well for lichen specimens. Although not as accurate as a high-fidelity polymerase, MyFi comprises a proofreading component that allows a 3.5× higher fidelity than Taq DNA polymerase.

In addition to polymerase misincorporation, chimera formation is another common PCR-induced source of error. Low starting template concentration and low amplification cycle numbers tend to reduce the rate of chimera formation (Lahr and Katz 2009). Here, 25 cycles were used for both amplifications, and no chimeras were detected using the LLA pipeline. However, after manual inspection of the sequences obtained, several chimeric sequences (about 3%) were detected. Upon closer inspection, these chimeras were found to be formed of concatenated sequences of the full-length amplicon, together with primer sequences and barcodes. These concatemers have previously been reported in SMRT amplicon sequencing studies (e.g., Jones and Kustka 2017), and they are thought to be generated not during the PCR steps, but during the SMRTbell adaptor ligation step of the PacBio library preparation (Fichot and Norman 2013). They can readily be discarded by screening sequences of uncharacteristically large size (Jones and Kustka 2017).

Metagenomics and metabarcoding studies using two-step PCR protocols often include triplicate samples for amplification, which are then pooled and used as a template for the second PCR (e.g., Schlaeppi et al. 2016; Schloss et al. 2016). In community analysis, pooling triplicates is thought to be useful in decreasing PCR bias introduced by inherent differences or stochastic fluctuations in amplification efficiencies (Kennedy et al. 2014), although no study has confirmed that it influences the results of community analyses significantly (Kennedy et al. 2014; Smith and Peay 2014). Using replicates can also reduce the impact of PCR failure due to pipetting-related issues, but it is more expensive and time-consuming. Here, a ‘cherry-picking’ approach was used where negative samples were redone using a different genomic DNA concentration. Although additional positive samples were recovered with this approach, it may still be possible that PCR product could be recovered from the remaining negative samples using replicates and a broader range of genomic DNA concentrations. Due to large amounts and high diversity of secondary compounds in lichen specimens and their generally low genomic DNA yields, the window of concentration at which amplification is successful is often very narrow, and PCR results are sometimes difficult to reproduce. Using several dilutions of each genomic DNA extract and pooling their products after the first PCR could therefore help in maximising the number of positive samples.

### Long Amplicon Analysis and the recovery of high-quality sequences for the target lichenised fungal species

The Long Amplicon Analysis pipeline has been developed by PacBio to phase and detect alleles in diploid organisms, and enables differentiation between underlying sequences that are 99.9% similar, such as haplotypes and pseudogenes (Bowman et al. 2014; Lleras et al. 2014). In this study, as an alternative to cloning, the LAA pipeline was used to recover high-quality barcode sequences from mixed DNA samples of target and non-target fungal species occurring in lichen specimens. The LAA pipeline is accessible on the SMRT portal and can be run by the sequencing service provider together with the primary analysis. The output data generated by LAA included fasta and fastq files with all sequence variants, as well as accuracy and coverage values for each of these sequences. Sequences with the highest coverage generally corresponded to the target species and for more than half of the samples, one single sequence was recovered for the target species. For the other samples, 2–8 sequence versions were recovered per target species. Most often the ITS variation within sample was caused by low quality consensus sequences, which could then be identified due to their low coverage. Occasionally, however, several versions of the ITS barcode with high coverage were recovered within a sample. The percentage identity between these versions mostly ranged between 95–99%. The presence of multiple versions is likely the result of sample heterogeneity (more than one target individual per sample), although carry-over contamination cannot be excluded. Additionally, intragenomic heterogeneity (several ITS copies per nucleus) and intramycelial heterogeneity (several nuclei per mycelium), as discussed in detail in Mark et al. (2016), are two other possible biological reasons for sequence variation. Other ITS sequences obtained corresponded to co-amplifying micro-organisms, predominantly fungi. The most common co-amplified fungi were from the orders Chaetothyriales (Eurotiomycetes) and Capnodiales (Dothideomycetes). The identity of these co-amplified fungi suggested two possible origins for these sequences: either the fungi occur naturally within the lichen thallus (endolichenic fungi), or lichenised or non-lichenised fungal species occur adjacent to the target species on the same substrate, and were accidentally co-sampled during the preparation of material for DNA extraction.

Most target sequences had high accuracy and coverage values, and a subset of these was validated with Sanger sequences obtained from the same DNA extracts. Initially, the size variation among amplicons recovered was more than the 10% recommended by PacBio and was of potential concern. An excess of sequences for the shorter amplicons relative to the longer ones could have been problematic, because lichenised fungi often have long ITS regions due to the presence of introns. However, at this low level of multiplexing (one marker for 96 samples), the difference in amplicon size did not prevent the generation of high-quality sequences for the target species. Moreover, at a higher multiplexing level (5 genes for 96 samples), the sequencing bias due to amplicon size variation did not seem to influence the results (Chen et al. 2015).

A recent study identified some problems with the LAA pipeline, including the formation of a few incorrect or truncated sequences even at relatively high read depths (Francis et al. 2018). As a result, a new pipeline (C3S-LAA) was developed by these authors which differs from LAA by comparing similarity based on CCSs as opposed to uncorrected reads before the start of the clustering phase. Their new approach, which was used for the SMRT sequencing of long amplicons (4000–8000 bp), successfully eliminated these incorrect and truncated sequences (Francis et al. 2018). We have not observed these problems with our data. However, LAA did not detect chimeras that were formed by concatemers of amplicons with primers and barcodes, sometimes with the second sequence being the reverse complement of the first (“siamaeras”, Hackl et al. 2014). However, these concatemers are easily detected with a BLAST comparison and filtered out because of their large size. In addition, some reverse complement sequences (or sequences with slightly truncated barcodes) were not recognised as being identical to other sequences by LAA and were therefore attributed to different clusters. This did not prevent LAA from recovering high-quality sequences for most target species, but it did add some time and effort in verifying whether or not they corresponded to true variants.

## Conclusion

PacBio amplicon sequencing is a promising approach for the metabarcoding of lichen specimens, and can be applied to the generation of reference sequences and the characterisation of lichen-associated fungal communities. Although restricted to specimens for which the genomic DNA is not overly degraded, this approach succeeded in generating full-length and high-quality ITS barcodes for specimens up to 25 years old. By scaling up the multiplexing level, this approach could significantly reduce the cost of barcode/sample and compete with Sanger sequencing as well as other NGS approaches.

## Supplementary materials

Supplementary material 1 Table S1 Data type: measurement This dataset is made available under the Open Database License (http://opendatacommons.org/licenses/odbl/1.0/). The Open Database License (ODbL) is a license agreement intended to allow users to freely share, modify, and use this Dataset while maintaining this same freedom for others, provided that the original source and author(s) are credited.Cécile Gueidan, John A. Elix, Patrick M. McCarthy, Claude Roux, Max Mallen-Cooper, Gintaras Kantvilas
